# Cold Atmospheric Helium Plasma in the Post-COVID-19 Era: A Promising Tool for the Disinfection of Silicone Endotracheal Prostheses

**DOI:** 10.3390/microorganisms12010130

**Published:** 2024-01-09

**Authors:** Diego Morais da Silva, Fellype Do Nascimento, Noala Vicensoto Moreira Milhan, Maria Alcionéia Carvalho de Oliveira, Paulo Francisco Guerreiro Cardoso, Daniel Legendre, Fabio Gava Aoki, Konstantin Georgiev Kostov, Cristiane Yumi Koga-Ito

**Affiliations:** 1 Institute of Science and Technology, São Paulo State University (UNESP), São José dos Campos 12227-010, SP, Brazil; diego.m.silva@unesp.br (D.M.d.S.); milhan.noala@gmail.com (N.V.M.M.); macoliveira12@gmail.com (M.A.C.d.O.); 2 Faculty of Engineering, São Paulo State University (UNESP), Guaratinguetá 12516-410, SP, Brazil; fellype@gmail.com (F.D.N.); konstantin.kostov@unesp.br (K.G.K.); 3 Division of Thoracic Surgery, Instituto do Coração, Hospital das Clínicas HCFMUSP, Faculdade de Medicina, Universidade de São Paulo, São Paulo 01246-903, SP, Brazil; cardosop@gmail.com; 4 Adib Jatene Foundation, Dante Pazzanese Institute of Cardiology, São Paulo 04012-909, SP, Brazil; daniel@fajbio.com.br; 5 Institute of Science and Technology, Federal University of São Paulo (UNIFESP), São José dos Campos 12231-280, SP, Brazil; fgaoki@unifesp.br

**Keywords:** multispecies biofilm, non-thermal plasma, endotracheal tubes, silicone prosthesis, COVID-19

## Abstract

Despite the excellent properties of silicone endotracheal prostheses, their main limitation is the formation of a polymicrobial biofilm on their surfaces. It can cause local inflammation, interfering with the local healing process and leading to further complications in the clinical scenario. The present study evaluated the inhibitory effect of cold atmospheric plasma (CAP) on multispecies biofilms grown on the silicone protheses’ surfaces. In addition to silicone characterization before and after CAP exposure, CAP cytotoxicity on immortalized human bronchial epithelium cell line (BEAS-2B) was evaluated. The aging time test reported that CAP could temporarily change the silicone surface wetting characteristics from hydrophilic (80.5°) to highly hydrophilic (<5°). ATR-FTIR showed no significant alterations in the silicone surficial chemical composition after CAP exposure for 5 min. A significant log reduction in viable cells in monospecies biofilms (log CFU/mL) of *C. albicans*, *S. aureus*, and *P. aeruginosa* (0.636, 0.738, and 1.445, respectively) was detected after CAP exposure. Multispecies biofilms exposed to CAP showed significant viability reduction for *C. albicans* and *S. aureus* (1.385 and 0.831, respectively). The protocol was not cytotoxic to BEAS-2B. CAP can be a simple and effective method to delay multispecies biofilm formation inside the endotracheal prosthesis.

## 1. Introduction

Tracheal stenosis is a clinical condition characterized by a reduction in the central airway diameter through a congenital or acquired pathological process. The COVID-19 pandemic produced a high number of patients submitted to intubation and prolonged mechanical ventilation worldwide, resulting in higher percentages of laryngotracheal stenosis [1,2,3,4]. Commonly, patients with tracheal stenosis after COVID-19 cannot undergo tracheal resection since maturation of the stenosis, as well as a reduction in inflammation, needs to be achieved before the procedure [5]. Additionally, the recurrence of tracheal stenosis is a risk during the first three months after surgery [6]. The prevalence of post-intubation tracheal stenosis in retrospective studies ranges from 6% to 20% and that of post-tracheostomy from 0.6% to 21% [7].

Patients with benign tracheal stenosis or tracheal tumors who are not eligible, either temporarily or permanently, for definitive treatment via tracheal surgical resection are considered for airway stenting to maintain airway patency. Silicone airway stents are widely used for their safety, patient tolerance, easy handling, and lower cost than that of self-expanding stents. The silicone T-tube was introduced in 1965 [8]. It requires a tracheostomy for anchoring the horizontal limb of the prosthesis that is occluded by a removable silicone cap. The straight-studded silicone stent was introduced in 1990 [9] and does not require a tracheostomy. Both are used as endoluminal support for the trachea, enabling breathing and phonation through the natural airway, thus improving quality of life [10].

Medical-grade silicone (MGS) is the primary material in endotracheal prostheses. MGS has attractive properties such as chemical inertness, water and temperature resistance, biocompatibility, and flexibility [11]. The constant contact between the inhaled air passing through the airway prosthesis and the tracheal underlying mucosa promotes the accumulation of biofilms composed of bacteria and fungi. The interaction between the polymicrobial biofilm and the surrounding mucosa can potentially aggravate the severity of the stenosis [10,12,13]. *Staphylococcus aureus*, *Pseudomonas aeruginosa*, and *Candida albicans* are the main microorganisms in the polymicrobial biofilm formed inside the patient’s silicone prosthesis implant [14]. Biofilm formation usually takes approximately seven days, and severe complications such as pneumonia or sepsis can occur [15]. On the other hand, the MGS degradation promoted by the polymicrobial biofilm is progressive, requiring the prosthesis to be changed every 6 to 12 months after implantation. This can negatively impact the patient’s quality of life and induce higher costs [16,17]. 

Previous studies focused on modifying the MGS surface to promote antimicrobial activity. Silver ions and nanoparticles were used as an alternative approach and were effective against *Escherichia coli*, *P. aeruginosa*, and *Staphylococcus aureus*. The limitations of these methods are the potential cytotoxicity and mucus accumulation on the silver-coated surface, which can reduce its antimicrobial properties [18]. 

Cold atmospheric plasma (CAP) stands for a low-temperature (<40 °C) gas discharge plasma with extensive applications in the medical and biomedical fields [19,20,21,22]. The literature has already reported microbial inactivation due to the reactive oxygen and nitrogen reactive species (RONS) and UV radiation produced by CAP. The antimicrobial effect was observed on several species of bacteria and fungi, including *S. aureus*, *P. aeruginosa*, and *C. albicans* [23,24,25]. CAP can be delivered through long, flexible plastic tubes [26,27], enabling clinical use. Moreover, previous studies reported that CAP has low toxicity to mammalian cells [28,29]. 

This pre-clinical in vitro study focused on the effect of the Helium-CAP over monospecies and multispecies biofilms grown on the MGS surface in a laboratory setting, which mimics what is clinically observed inside the T-tube. The CAP treatment’s effects on biofilms’ viability were assessed, along with the possible surface changes on the MGS caused by CAP. Lastly, the cytotoxicity of the protocol was tested using a BEAS-2B immortalized human bronchial epithelium cell line. The motivation of this study is to develop a protocol for CAP treatment that can be applied directly to the external limb of the T-tube during outpatient visits, aiming to control the biofilm proliferation in the lumen of the silicone prosthesis. Such features can prolong the prosthesis’ durability, reduce the interaction between the biofilm and the adjacent mucosa, and enhance the local healing process.

## 2. Materials and Methods

The MGS samples were prepared in disks measuring 8 mm in diameter and 2 mm in height. The specimens had the same chemical composition as T-tube implants. They were used to perform the surface characterization of silicone before and after the plasma treatment and the microbiological assays. Before treatment, all samples were washed in an ultrasonic bath by immersion for 10 min in water and 10 min in isopropyl alcohol. After that, another step of water washing was performed for 10 min. Finally, the samples were sterilized in an autoclave for 20 min packed in a medical-grade sheet. The MGS samples were stored in a dry place until the experiments.

### 2.1. Characterization of CAP Interactions with the MGS Surface

**Figure 1:**
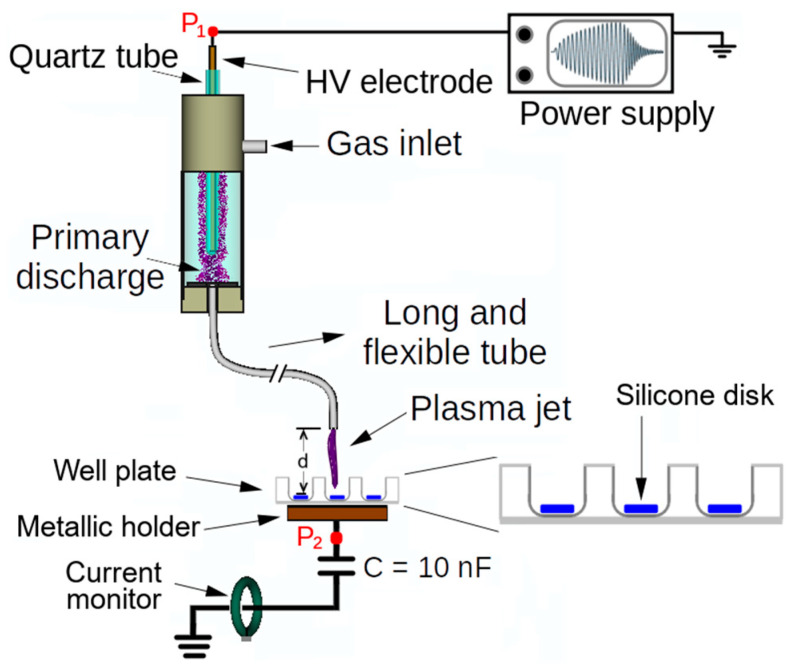
Scheme for CAP generation and MSG treatment.

Figure 1 illustrates the configuration of the CAP system used to treat MGS samples. The device was described in [24]. It mainly consists of a dielectric barrier discharge (DBD) type reactor, composed of a metallic pin electrode placed inside a closed-end quartz tube, which, in turn, is placed inside a dielectric enclosure. The working gas (helium, 99.2% purity) is fed into the chamber and flushed to the ambient air through a 1 m long and flexible plastic tube connected to the reactor output (inner and outer diameter equal to 2.0 mm and 4.0 mm, respectively). A copper wire with a diameter of 0.5 mm was installed inside the plastic tube and placed a few millimeters inside the reactor to avoid contact with the quartz tube. The other tip of the copper wire terminates 2 mm before the output tip of the plastic tube. The high voltage is turned on when the working gas flows, and a primary discharge is ignited inside the DBD reactor. The latter polarizes the copper wire, and a small plasma jet is ignited at the end of the plastic tube.

A commercial AC generator from GBS Elektronik GmbH (model Minipuls4) was used as the power source to generate the CAP. It was used to produce an amplitude-modulated high-voltage (HV) waveform that consists of a sinusoidal “burst” with an oscillation frequency of 31.7 kHz, followed by a voltage-off interval, which repeats at a repetition period (T) equal to 1.2 ms. Such an AC generator was chosen due to its versatility in setting up the main operating parameters (voltage waveform, amplitude, and frequency), allowing us to test different combinations. In previous studies, we have found the operating parameters that do not heat up the target and provide good antimicrobial efficacy [25]. The discharge power for the configuration shown in Figure 1 was calculated by measuring the HV input at point P1 and the voltage on the capacitor at point P_2_ [24]. Thus, the discharge power obtained in the current configuration was 436 ± 2 mW.

MGS samples were placed inside a 24-well plate at a distance d = 5.0 mm from the plasma outlet. The employed gas flow rate was 2.0 SLM. Aiming to study the interaction between the CAP and the MGS, as well as the behavior of the MGS surface after 2 min of plasma exposure, the CAP was generated by applying HV with a peak-to-peak amplitude of 17.2 kV to the pin-electrode, which is nearly 40% higher than the value employed for the treatment of biofilms. This value was chosen to simulate an extreme case and evaluate the possibility of material damage due to plasma exposure. The samples were characterized by wettability measurements and attenuated the total reflectance mode of Fourier transform infrared spectroscopy (ATR-FTIR) before and after CAP treatment. To assess the water contact angle (WCA), the samples were analyzed using an F300 Rame-Hart goniometer (Rame-hart, Washington, DC, USA). Six WCA measurements were performed, one before plasma exposure and five after the treatment (at time instants of 0 min, 15 min, 30 min, 1 h, and 2 h). Due to the small sample size, different samples were used for each WCA measurement. To analyze possible changes in the surface chemistry of the MGS samples exposed to CAP treatment, ATR-FTIR spectra were recorded before and right after the CAP exposure. Such measurements were carried out using the ATR mode of a Lambda-100 spectrometer (Perkin-Elmer, Shelton, CT, USA) in the region between 4000 and 400 cm^−1^, with a 4 cm^−1^ resolution averaged over 16 scans.

### 2.2. Formation of Monospecies Biofilms on MGS Surfaces

Reference strains (Table 1) were plated in Brain Heart Infusion (BHI) agar for bacteria or Sabouraud agar for fungi. The plates were incubated for 24 h at 37 °C under aerobiosis. After, standardized suspensions (1 × 10^7^ CFU/mL) were prepared in sterile saline (NaCl 0.9%) with the aid of a spectrophotometer (AJX-1600, Micronal, São Paulo, SP, Brazil). The optical density and the wavelength adopted for each microorganism are shown in Table 1. Sterile MGS specimens were transferred to a 24-well plate, and 2.0 mL of brain heart infusion (BHI) broth and 200 µL of the microbial inoculum were added to each well. The plates were incubated for 48 h at 37 °C under aerobiosis and agitation (120 rpm). The culture medium was refreshed after 24 h. 

### 2.3. Formation of Multispecies Biofilms on MGS Surfaces

The microbial suspensions were obtained as described in Section 2.2. The multispecies biofilms were grown on the surface of MGS inside a 24-well plate. In total, 2.0 mL of BHI broth + 2.5% Bovine Serum Albumin (BSA) were added to each well. Then, 200 µL of the *C. albicans* and *S. aureus* inoculum were added, followed by 20 µL of *P. aeruginosa* inoculum. The plates were incubated for 48 h at 37 °C under aerobiosis and agitation (120 rpm). The culture medium was refreshed after 24 h.

### 2.4. CAP Treatment of the Biofilms Formed on MGS

**Figure 2:**
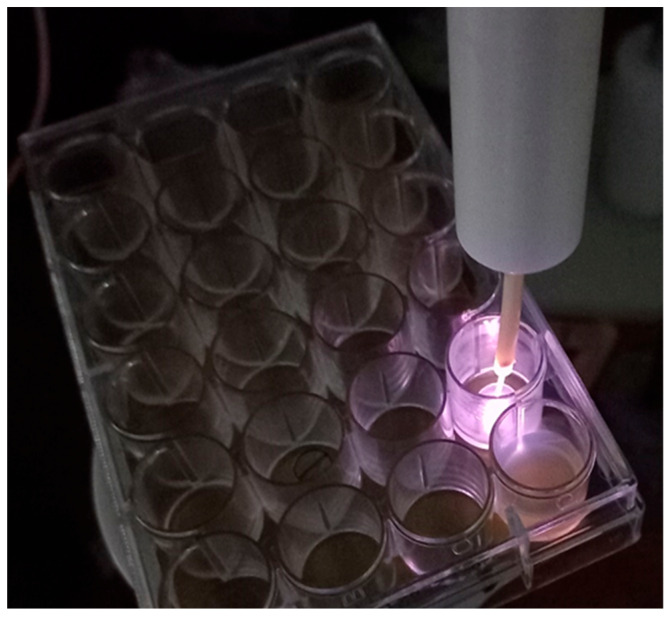
Application of the cold atmospheric plasma jet on the sample surface inside a well (24-well plate), with a distance of 0.5 cm between the plasma outlet and the sample.

Each well containing the biofilms was separately exposed to He-CAP treatment for 5 min. CAP was produced by the device described in Figure 1 under the same conditions employed to study the interaction between the plasma and the MGS, with a voltage amplitude of 12.3 kV p-p. For the CAP treatment, each sample was placed exactly in the geometrical center of the well (from a 24-well plate), as seen in Figure 2. The distance between the sample surface and the CAP output was 0.5 cm.

Biofilms were also flushed via He gas flow without plasma ignition for comparative purposes.

### 2.5. Determination of Viable Cell Counts

After the CAP exposure, the biofilms were recovered from the MGS specimens via sonication (amplitude of 50%) in 3 cycles of 10 s pulse on, intercalated with 20 s pulse off. Finally, the suspensions were serially diluted and plated in specific agar to determine viable cell counts (CFU/mL), according to Table 2. The experiments were performed in triplicate on three independent occasions (*n* = 9). The data obtained were analyzed statistically using the Shapiro–Wilk normality test and compared using the Mann–Whitney test. The level of significance was set at 5%.

### 2.6. Cytotoxicity Analysis

The cell line BEAS-2B was selected to be used in this study to evaluate the toxicity of the CAP exposure protocol on the human bronchial epithelium. The cytotoxicity analysis was based on ISO 10993-5/2009 [30]. The cells were incubated in Gibco^TM^ LHC-9 medium (Thermo Fisher, Waltham, MA, USA) and kept incubated at 37 °C and 5% CO_2_ until they reached the desired confluence. To evaluate the cytotoxicity, 4 × 10^4^ cells per well were plated in 24-well plates. The experiments were performed in duplicate (*n* = 12). For the treatment, 200 µL of the new medium was added to the wells to prevent them from drying out, and the cells were exposed to the products of CAP generated inside the T-tube. 

**Figure 3:**
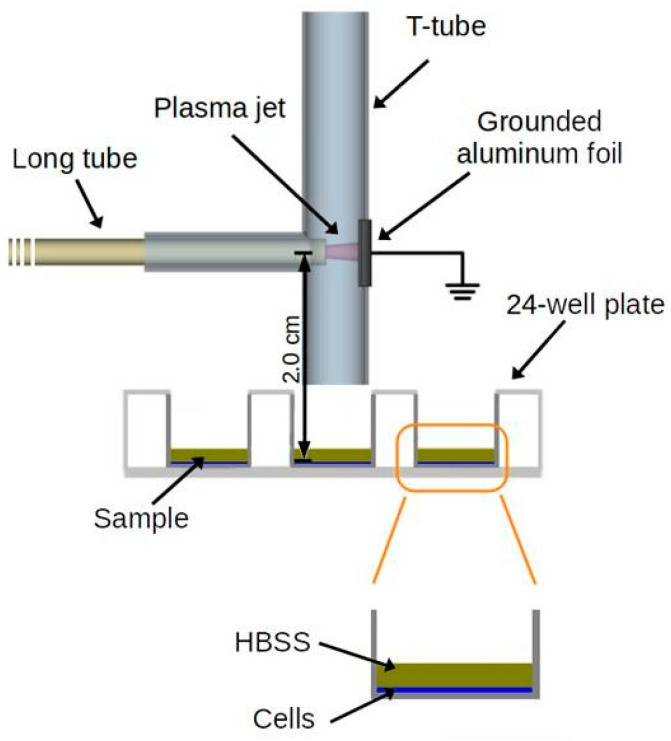
Schematic of the experimental setup to generate CAP inside the T-tube during the cytotoxicity test.

Figure 3 shows the schematic for producing and applying the He CAP inside the tracheal T-tube. For this assay, the flexible tube from the CAP device was inserted horizontally inside the extratracheal portion. The 24-well plate was positioned under the T-tube vertical portion so that the distance between the CAP generated inside the T-tube and the cells was 2.0 cm. This configuration was used to study the effects of the CAP products on BEAS-2B cells. The treatment parameters were 31.7 kHz of frequency, 12.3 kV of voltage amplitude, and 2.0 SLM of helium gas flow, and the treatment group was exposed to CAP for 5 min. In the control group, the cells were not exposed to plasma. 

After CAP exposure of each well, 1 mL of the new medium was added to the wells. After 24 h, the medium was removed, the MTT reagent ((3-4,5-dimethylthiazol-2yl)-2,5-diphenyl-tetrazolium, Sigma, St. Louis, MI, USA) was added, and the plates were kept in agitation for 10 min. The optical density of the resulting solution was measured using a spectrophotometer at 570 nm. The control group normalized the obtained absorbance (=100%). The obtained data were evaluated in the GraphPad Prism software, version 8 (GraphPad Software, Inc., La Jolla, CA, USA). Cytotoxicity classification was based on the cell viability percentage, where values above 70% were considered non-cytotoxic [31]. 

### 2.7. Statistical Analysis

The collected data were statistically evaluated using GraphPad Prism version 8 (GraphPad Software, Inc., La Jolla, CA, USA). The normal data distribution was evaluated, and the most appropriate statistical tests were selected and applied. The significance level of 5% was adopted for all the tests.

## 3. Results

### 3.1. MGS Surface Characterization after CAP Exposure

**Figure 4:**
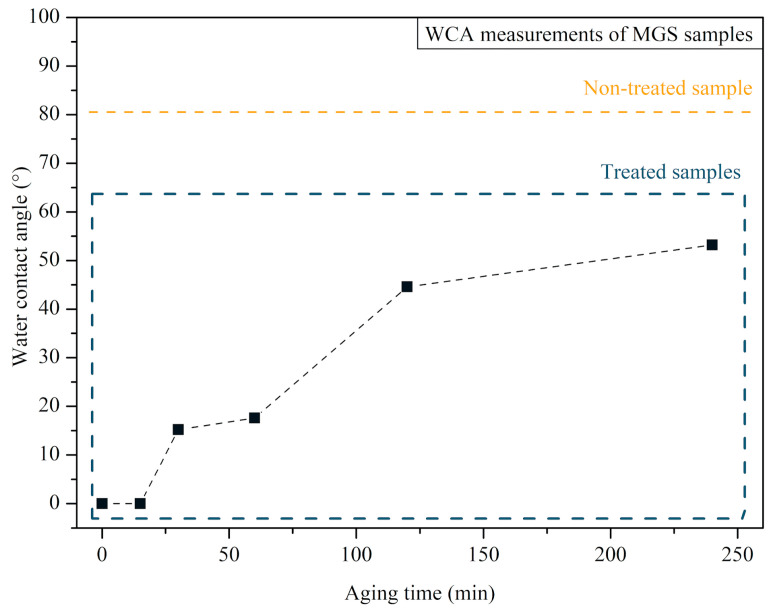
Aging time test of MGS samples after the treatment of CAP. The period of analysis varies between 0 min and 4 h. The WCA of the non-treated sample is displayed in a box inside the graphic for comparison purposes.

**Figure 5:**
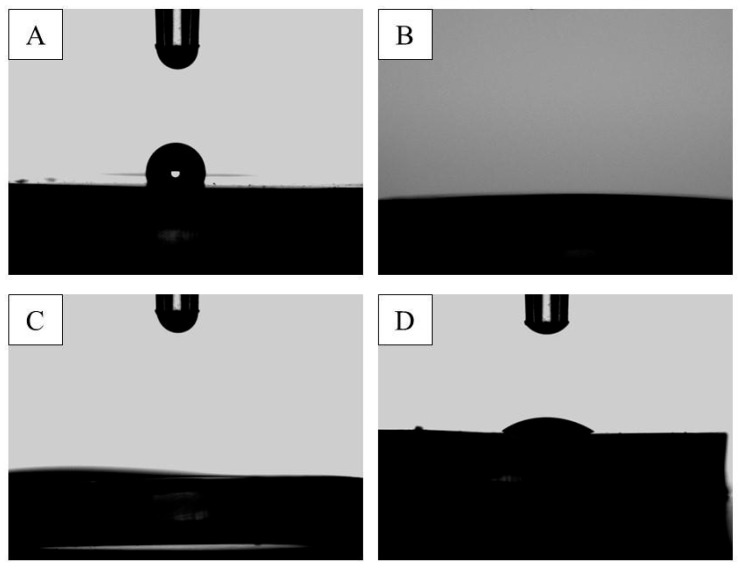
WCA measurement images showing the behavior of MGS surfaces in contact with water droplets at different times: (**A**) before CAP exposure; (**B**) right after CAP exposure; (**C**) 15 min after CAP exposure; (**D**) 4 h after CAP exposure.

The WCA behavior measured on the surface of the MGS samples after exposure to CAP treatment is presented in Figure 4. Images of the water droplets on the MGS samples are shown in Figure 5. Non-treated specimens have a WCA of 80.5°, i.e., exhibiting a less hydrophilic surface when compared to the control. The sample analyzed immediately after the CAP exposure showed a WCA < 5°, indicating that CAP changed the MGS surface properties to a highly hydrophilic surface. The MGS surface characteristics after CAP exposure, that is, WCA < 5°, were maintained for approximately 15 min. After 30 min, the WCA increased to 15.2°, and this increasing trend was also observed for the sample analyzed after one hour, two hours, and four hours, showing a WCA of 17.6°, 44.6° and 53.2°, respectively.

**Figure 6:**
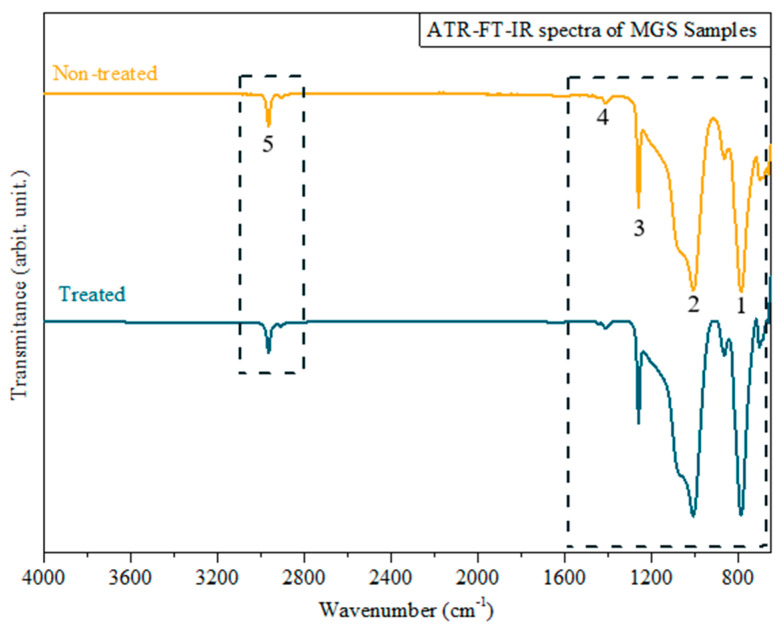
ATR-FTIR spectra of treated (blue) and non-treated (yellow) MGS samples. Numbers 1 and 2 represent –CH_3_ of SiCH_3_ bands; 3 and 4 represent Si–O–Si bands; and 5 is related to C–H bands.

Figure 6 shows the spectra obtained from the ATR-FTIR analysis of non-treated and CAP-treated samples. This analysis aimed to look for possible changes in the MGS compositional properties before and after plasma irradiation. Table 3 contributes to the spectra analysis, presenting the correlation between the detected bands and the main functional groups on the MGS surface. The spectra of both samples demonstrate the C-H stretching band at 2962.3 cm^−1^, the asymmetric stretching of methyl groups at 1412 cm^−1^ characteristic for Si-CH_3_, and the symmetric stretching of the same group at 1258 cm^−1^. Finally, the typical bands at 800–1000 cm^−1^ are attributed to the Si-O-Si bond stretching [32].

### 3.2. Antimicrobial Activity of CAP

**Figure 7:**
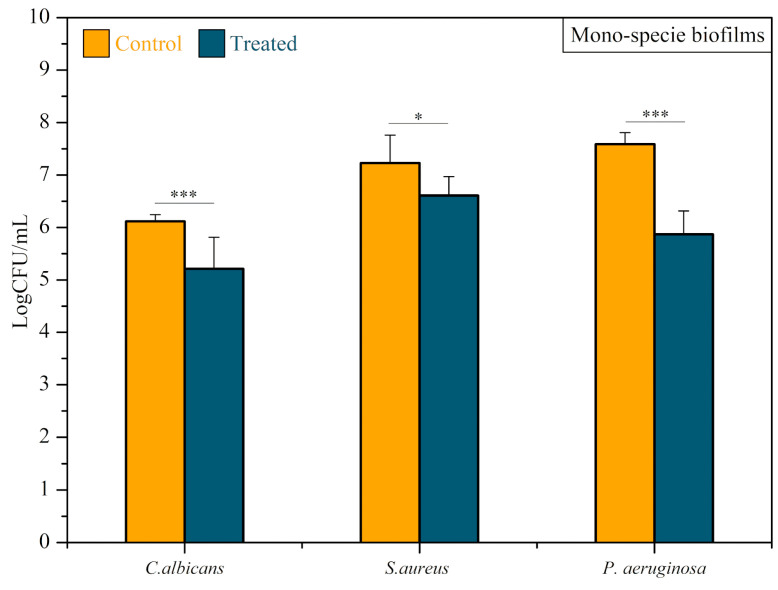
Antibiofilm efficacy of CAP on *Candida albicans*, *Staphylococcus aureus*, *and Pseudomonas aeruginosa* monospecies biofilms. Results expressed in the logarithm of colony-forming units per milliliter (log CFU/mL). *T*-test *** *p* < 0.0001 and * *p* < 0.05.

The effect of CAP on the monospecies biofilms of *C. albicans*, *P. aeruginosa*, and *S. aureus* formed on the MGS surface can be observed in Figure 7. The *C. albicans* CAP-treated group demonstrated an average count of viable cells of 1.362 × 10^6^ (CFU/mL), while the control group was 3.151 × 10^5^ (CFU/mL). The treatment resulted in a statistically significant log reduction of 0.636 (*T*-test, *p* < 0.0001). The plasma-treated *P. aeruginosa* group presented an average count of viable cells of 1.42 × 10^6^ (CFU/mL), while the average value of the control group was 3.955 × 10^7^ (CFU/mL). In this way, there was a statistically significant log reduction of 1.445 (Mann–Whitney test, *p* < 0.0001). *S. aureus* monospecies biofilm counts of viable cells of 5.194 × 10^6^ (CFU/mL) and 2.844 × 10^7^ (CFU/mL) were observed for treated and control groups, respectively, with a statistically significant log reduction of 0.738 (*T*-test, *p* < 0.05).

**Figure 8:**
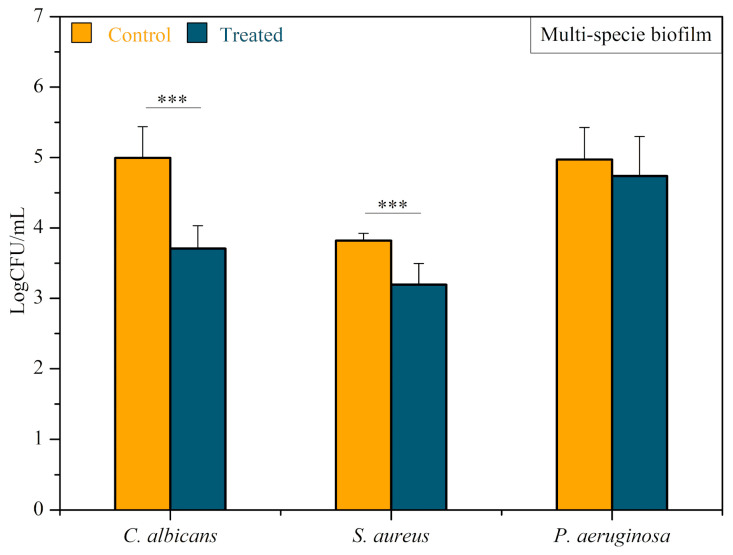
Antibiofilm efficacy of CAP on *C. albicans*, *P. aeruginosa*, *and S. aureus* multispecies biofilms. Results are expressed in the logarithm of colony-forming units per milliliter (CFU/mL). Mann–Whitney test, *** indicates *p* < 0.0001.

Multispecies biofilms composed of *C. albicans*, *P. aeruginosa*, *and S. aureus* were also treated with CAP. Figure 8 shows the obtained results. The average count of *C. albicans* viable cells recovered from plasma-treated biofilm was 6.333 × 10^3^ (CFU/mL), while that from multispecies biofilm control was 1.571 × 10^5^ (CFU/mL), indicating a statistically significant log reduction of 1.358 (Mann–Whitney test, *p* < 0.0001). Plasma-treated *P. aeruginosa* biofilms had 1.551 × 10^5^ (CFU/mL) viable cells, while 1.655 × 10^5^ (CFU/mL) viable cells were recovered from the control biofilm. Thus, *P. aeruginosa* showed a slight log reduction of 0.028 (*p* > 0.05). The average value of *S. aureus* viable cells recovered from the plasma-treated and the control groups was 1.0 × 10^3^ (CFU/mL) and 6.77 × 10^3^ (CFU/mL), respectively. There was a statistically significant log reduction of 0.83 (Mann–Whitney test, *p* < 0.0001).

### 3.3. Cytotoxicity Test

**Figure 9:**
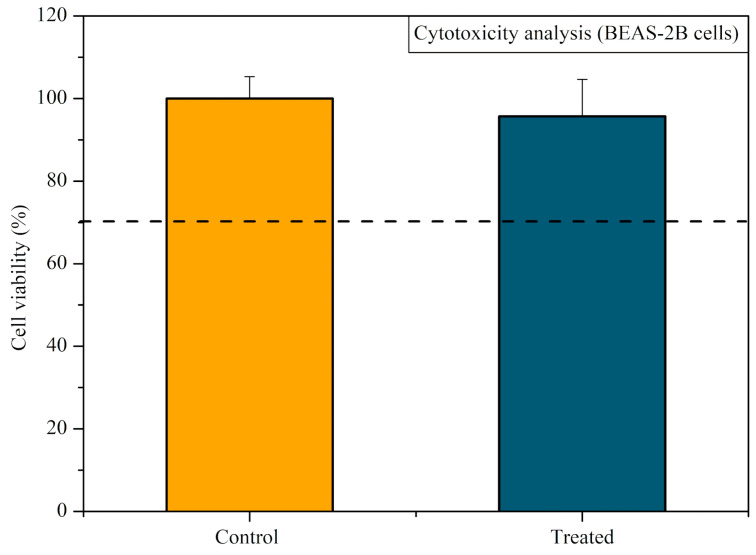
Cytotoxicity analysis of CAP expressed in cell viability (control 100%). The BEAS-2B cells were exposed to CAP generated inside the T-tube for 5 min. The cell viability was determined 24 h after CAP exposure. The dashed line represents 70% of cell viability.

The results of the cytotoxicity test using the BEAS-2B cells are presented in Figure 9. The viability of the cells exposed to CAP generated inside the T-tube was 95.76%. Thus, the protocol with antimicrobial activity can be considered non-cytotoxic (viability > 70%). 

## 4. Discussion

The increase in tracheal stenosis cases related to previous episodes of COVID-19 emerged as an alarming reality after the pandemic. Beyouglu et al. (2022) reported that the intubation time of patients with COVID-19 is significantly longer when compared to that of patients with other conditions, which further increases the occurrence of tracheal stenosis and the subsequent use of silicone endotracheal prostheses [34]. To avoid the prostheses changing every 6 to 12 months after implantation, alternative treatments that do not negatively interfere with the properties of MGS and present disinfection properties are needed. This study investigated CAP as an antimicrobial tool for removing biofilm commonly found in silicone endotracheal prostheses. 

In the first stage, MGS surface characterization was performed after CAP exposure. The WCA increased steadily with time after the CAP treatment. In contrast, in the period studied, it did not recover to the same WCA before CAP exposure (80.5°). Thus, the plasma treatment temporarily changes the MGS surfaces, making them highly hydrophilic; halfway through the treatment, the WCA increases but does not reach the same contact angle as the non-treated sample. After the treatment, the MGS surface at 4 h is still more hydrophilic than the untreated MGS.

Regarding the ATR-FTIR measurements, comparing both spectra, no significant differences were observed in the treated MGS sample compared to the untreated one. This is a positive result, as it shows that the plasma interacts with the MGS material, changing its surface energy without causing any change in its chemical composition or structure. 

The hydrophobicity changes highlighted in the WCA analysis can be attributed to the interaction between the MGS surfaces and the RONS produced by CAP [35], leading to the formation of polar groups. Conversely, the ATR-FTIR analysis did not detect these plasma-induced polar groups. However, we should consider that when an internal reflection element is used in ATR-FTIR analysis, the IR beam detects chemical groups to the depth of 0.5–5 µm [36]. So, suppose the plasma modification is limited only to the sample surface. In that case, the ATR-FTIR will probably not be able to detect any changes because it integrates the material response over a much broader depth. On the other hand, the behavior highlighted in the WCA aging test on the MGS surface occurs in the outermost surface layers (few nm) that were affected by the plasma. In this sense, further analyses more sensitive in detecting chemical changes on the MGS surfaces may clarify some of our findings.

To the best of our knowledge, the usage of CAP to treat biofilms on MGS surfaces at our set-up conditions has not been previously reported. However, biofilms treated with CAP on different substrates have already been investigated, and this will be a parameter to discuss our results. 

In a previous study, *S. aureus* (ATCC 33591) and *P. aeruginosa* (ATCC 27853) biofilms formed on collagen membranes and were treated with CAP for 5 min with 15 mm of working distance. The treatment against these biofilms was as effective as the treatment performed in our study. The authors observed higher *S. aureus* log reduction than our experiments for the same microorganism [37]. However, it is worth mentioning that we used a different *S. aureus* strain, which could justify some differences in the data. In this previous work, the reported log reduction for *P. aeruginosa* biofilm was lower than the decrease observed in our experiments for the same strain (ATCC 27853). These results indicate that reducing the working distance to 5 mm improves the antimicrobial effectiveness of *P. aeruginosa* biofilm. 

The findings in the literature regarding CAP treatment against *C. albicans*, using similar parameters, are available to ATCC 18804. Inhibition zones of 2.0 and 2.5 cm were reached after 150 s treatment [23]. Another study with *C. albicans* strain SC 5314 reported a 2-log reduction when CAP was applied using the same parameters used in the present study and 15 mm of working distance [38]. It is well known that this strain is very virulent, reinforcing the antifungal potential of CAP treatment, as well as its antibacterial effect. 

Our study also demonstrated the potential of CAP for treating multispecies biofilms, which more closely reflects the clinical situation of endotracheal prostheses. *P. aeruginosa* was the only microorganism that did not show a statistically significant reduction after the treatment of multispecies biofilms. This finding probably occurred due to *P. aeruginosa*’s dispersal in the polymeric extracellular matrices within *C. albicans* and *S. aureus*, with no or little interaction between *P. aeruginosa* and CAP reactive species. Similarly, the literature reports a high resistance of *P. aeruginosa* to different antibiotics, such as tobramycin and ciprofloxacin. This behavior can be attributed to the disposal of *P. aeruginosa* throughout the biofilm architecture due to the hypoxic and low metabolic conditions known as contributors to antimicrobial resistance [39]. The low reduction in viable *P. aeruginosa* cells recovered from the multispecies biofilm treatment with CAP may be due to the same reason. Once the three microorganisms are part of the same biofilm structure, the disposal of each throughout the biofilm architecture and the competition between them can create the same hypoxic environment, leading to low metabolic conditions, and possibly increasing the *P. aeruginosa* resistance to the CAP treatment. Sequential applications of CAP can be an alternative to enhance the antibacterial activity against *P. aeruginosa* in multispecies biofilms. As this study did not explore the sequential application of CAP, further studies are necessary to better understand its effects on *P. aeruginosa* in multispecies biofilms. 

In addition to being antimicrobial, the proposed protocol was not cytotoxic to tracheal cells (BEAS-2B) in the conditions of this study. We emphasize that we focused on exposing the MGS used in T-tubes to CAP and not directly to the cells, simulating a clinical situation where the CAP jet will be directed to the contaminated prosthesis. We observed that a controlled application of CAP in the prosthesis is unlikely to cause harm to the epithelial cells adjacent to and outside the wall of the T-tube. Future studies are necessary to clarify the distribution of RONs inside the tube and to check the improvement in antimicrobial activity after sequential exposures since promising findings have already been obtained after only one application. 

## 5. Conclusions

In conclusion, CAP exposure of MGS samples enhances the surface hydrophilic properties from hydrophilic (WCA = 80.5°) to highly hydrophilic (WCA < 5°). The plasma-jet-induced changes on the surface of the sample were not permanent. ATR-FTIR analysis did not show any significant differences between CAP-treated and non-treated samples, essentially indicating only a modification on the surface of the sample. CAP showed an effective inhibitory effect on all monospecies biofilms formed on MGS surfaces. It also demonstrated antimicrobial efficacy against *C. albicans* and *S. aureus* cells in the multispecies biofilm. The cytotoxicity test showed that the protocol is not cytotoxic for BEAS-2B cells when CAP is generated inside the T-tube. Future studies are required to better understand CAP’s potential and limitations in controlling the polymicrobial biofilms inside an endotracheal prosthesis and to make further improvements to the protocol. However, with the findings of this in vitro study, it is already possible to state that CAP is a suitable tool for the disinfection of MGS surfaces and, therefore, promising for the disinfection of silicone endotracheal prostheses. 

## Figures and Tables

**Table 1:** Data on the reference strains used for biofilm formation and parameters of optical density (O.D) and wavelength (λ) adopted for standardized suspension preparation (1 × 10^7^ CFU/mL).

Microorganism	Reference Number	Optical Density	Wavelength (nm)
*Candida albicans*	ATCC 18804	0.760	530
*Pseudomonas aeruginosa*	ATCC 27853	0.130	600
*Staphylococcus aureus*	ATCC 6538	0.249	490

**Table 2:** Description of each culture media used for viable cell recovery from mono- and multispecies biofilms.

	Microorganism	Culture Medium
Monospecies biofilm	*C. albicans*	Sabouraud agar
	*P. aeruginosa*	Brain Heart Infusion (BHI) agar
	*S. aureus*	Brain Heart Infusion (BHI) agar
Multispecies biofilm	*C. albicans*	CHROMagar
	*P. aeruginosa*	Cetrimide agar
	*S. aureus*	Mannitol agar

**Table 3:** Attribution to the bands identified in the ATR-FTIR analysis.

Functional Group	Wavenumber (cm^−1^)	Peak #	Reference
–CH_3_ of SiCH_3_	1258 and 1412	1, 2	[33]
Si–O–Si	800–1000	3, 4	
C–H	2962.3	5	

## Data Availability

The data that support the findings of this study are available from the corresponding author upon reasonable request.
